# Immune-related 3-lncRNA signature with prognostic connotation in a multi-cancer setting

**DOI:** 10.1186/s12967-022-03654-7

**Published:** 2022-09-30

**Authors:** Shimaa Sherif, Raghvendra Mall, Hossam Almeer, Adviti Naik, Abdulaziz Al Homaid, Remy Thomas, Jessica Roelands, Sathiya Narayanan, Mahmoud Gasim Mohamed, Shahinaz Bedri, Salha Bujassoum Al-Bader, Kulsoom Junejo, Davide Bedognetti, Wouter Hendrickx, Julie Decock

**Affiliations:** 1 College of Health and Life Sciences (CHLS), Hamad Bin Khalifa University (HBKU), Qatar Foundation (QF), Doha, Qatar; 2 Human Immunology Division, Research Branch, Sidra Medicine, Doha, Qatar; 3 Qatar Computing Research Institute (QCRI), Hamad Bin Khalifa University (HBKU), Qatar Foundation (QF), Doha, Qatar; 4 Present Address: St. Jude Children’s Research Hospital, Memphis, TN USA; 5 Translational Cancer and Immunity Center, Qatar Biomedical Research Institute (QBRI), Hamad Bin Khalifa University (HBKU), Qatar Foundation (QF), Doha, Qatar; 6 Department of Surgery, Leiden University Medical Center, Leiden, the Netherlands; 7 Women’s Hospital, Hamad Medical Corporation, Doha, Qatar; 8 Weill Cornell Medicine-Qatar, Doha, Qatar; 9 National Center for Cancer Care and Research (NCCCR), Hamad General Hospital, Doha, Qatar; 10 General Surgery Department, Hamad General Hospital, Doha, Qatar

**Keywords:** lncRNA, ICR, Immune checkpoint, Prognostic, Immune favorable

## Abstract

**Background:**

Advances in our understanding of the tumor microenvironment have radically changed the cancer field, highlighting the emerging need for biomarkers of an active, favorable tumor immune phenotype to aid treatment stratification and clinical prognostication. Numerous immune-related gene signatures have been defined; however, their prognostic value is often limited to one or few cancer types. Moreover, the area of non-coding RNA as biomarkers remains largely unexplored although their number and biological roles are rapidly expanding.

**Methods:**

We developed a multi-step process to identify immune-related long non-coding RNA signatures with prognostic connotation in multiple TCGA solid cancer datasets.

**Results:**

Using the breast cancer dataset as a discovery cohort we found 2988 differentially expressed lncRNAs between immune favorable and unfavorable tumors, as defined by the immunologic constant of rejection (ICR) gene signature. Mapping of the lncRNAs to a coding-non-coding network identified 127 proxy protein-coding genes that are enriched in immune-related diseases and functions. Next, we defined two distinct 20-lncRNA prognostic signatures that show a stronger effect on overall survival than the ICR signature in multiple solid cancers. Furthermore, we found a 3 lncRNA signature that demonstrated prognostic significance across 5 solid cancer types with a stronger association with clinical outcome than ICR. Moreover, this 3 lncRNA signature showed additional prognostic significance in uterine corpus endometrial carcinoma and cervical squamous cell carcinoma and endocervical adenocarcinoma as compared to ICR.

**Conclusion:**

We identified an immune-related 3-lncRNA signature with prognostic connotation in multiple solid cancer types which performed equally well and in some cases better than the 20-gene ICR signature, indicating that it could be used as a minimal informative signature for clinical implementation.

**Supplementary Information:**

The online version contains supplementary material available at 10.1186/s12967-022-03654-7.

## Background

Cancer treatment has radically changed over time, evolving from a one-size-fits-all approach to a more tailored, personalized approach. Furthermore, where once cancer treatment focused on the tumor the recent success of immunotherapy has highlighted the need to consider the tumor microenvironment in cancer care by harnessing the inherent anti-tumor immune response. Early clinical trials demonstrated the potential of immunotherapy to induce durable responses, resulting in immunotherapy being heralded as a turning point in cancer care. The first immune checkpoint inhibitor (ICI) against cytotoxic T-lymphocyte antigen number 4 (CTLA-4), ipilimumab, received FDA approval in 2011 for the treatment of advanced melanoma [1]. In the following years, the FDA approved the use of additional immune checkpoint inhibitors and extended their use for a range of tumor types based on their immune checkpoint ligand expression rather than their tissue-of-origin [1]. To date, immunotherapy has shown promising results in 15 different cancer types and the use of first-line treatment with the ICI pembrolizumab even outperforms conventional chemotherapy in a few cancer types [2, 3]. Unfortunately, the success of immunotherapy is limited to a minority of patients as a result of tumor intrinsic factors and microenvironmental modifiers, leading to a surge of studies aiming to identify immune-related gene signatures that could predict which patients would be more likely to benefit from immunotherapy.

In this study, we explored long non-coding RNA (lncRNA) profiles of tumors in relation to tumor immune phenotypes. The number and role of lncRNAs were previously underappreciated. Currently, the GENCODE project (v39) lists 18,811 human lncRNAs and 51,306 lncRNA transcripts, and lncRNAs have been involved in various biological processes regulating gene expression and post-transcriptional modification [4]. Furthermore, emerging evidence supports a role for lncRNAs in regulating the adaptive immune response in addition to the innate immune response with potential implications for cancer immunity and immunotherapy [5–7]. In particular, lncRNAs have been implicated in tumor immune escape through the regulation of the antigen presentation machinery as well as of immune cell development, recruitment and function [6–9]. In addition, few lncRNAs have been shown to modulate immune checkpoint expression, and hence may be associated with immunotherapy response [10, 11]. While a better understanding of the expression patterns and mechanistic roles of individual lncRNAs can help to dissect their biological functions in cancer, panels or signatures of lncRNAs will more likely hold prognostic and predictive potential. Various immune-related lncRNA signatures have been identified with prognostic connotations for specific cancer types, including gastric cancer, head and neck cancer, lung cancer, colorectal cancer and hepatocellular carcinoma [12–18]. In breast cancer, few lncRNA signatures have been associated with tumor immune infiltration or immune functional status [19–24]. Moreover, lncRNA-based immune-classification has been proposed to identify “immune-active” cases that are characterized by an immune-functional lncRNA signature, high T cell infiltration in tumors and improved immunotherapy benefit [25]. Together, these studies demonstrate the potential clinical value of immune-related lncRNA signatures, however, more studies with larger sample sizes and prospective study design are needed to validate these findings. Furthermore, the prognostic value of the reported signatures may be limited to the tumor type in which they were identified.

Here, we identified immune-related lncRNA signatures (and proxy protein-coding gene network) that are associated with clinical outcome and immune checkpoint expression in breast cancer and have prognostic value in multiple cancer types. Using the large TCGA breast cancer dataset, we first identified differentially expressed immune-related lncRNAs (ir-lncRNAs) in immune favorable versus immune unfavorable tumors as defined by the Immunologic Constant of Rejection (ICR), a prognostic gene signature of tumor immune activation [26–30]. Next, we mapped the ir-lncRNAs to a coding-non-coding gene network enabling the identification of proximal protein-coding genes using the random walk with restart (RWR) computational algorithm. We then investigated the biological role of these proximal protein-coding genes through pathway enrichment analysis. Finally, we identified a set of three ir-lncRNAs that are in addition associated with immune checkpoint expression and show a stronger effect on overall survival in multiple cancer types as compared with the ICR signature, highlighting the potential role of lncRNAs in defining the immune contexture of tumors.

## Methods

### Patient cohorts

Initial lncRNA analysis was performed using the TCGA breast cancer cohort, and identified lncRNA signatures were validated in several TCGA cancer datasets (BRCA [n = 798], HNSC [n = 417], SKCM [n = 216], UCEC [n = 311], LIHC [n = 191], STAD [n = 247], BLCA [n = 248], CESC [n = 190], KICH [n = 65], OV [n = 249], LUSC [n = 202], READ [n = 44], COAD [n = 112], LUAD [n = 469], GBM [n = 150], KIRP [n = 188], KIRC [n = 298], LGG [n = 478]) as well as a small breast cancer cohort from Qatar (RAQA [n = 24]) [31]. Clinical information and mRNA sequencing data from the TCGA datasets were obtained through the GDC portal as previously described [31], whereas lncRNA expression data was extracted from the TANRIC database.

RNA isolation and total RNA sequencing of the RAQA breast tumors was performed as previously reported [31]. Both gene and lncRNA expression data were subjected to quality control using FastQC (python v.2.7.1, FastQC v.0.11.2), adapter sequences were trimmed using flexbar (v.3.0.3), and reads were aligned to GRCh37 using hisat2 (v.2.1.0) and SAMtools (v.1.3). After alignment, QC was performed to verify the quality of the alignment and paired-end mapping overlap using Bowtie2 (v.2.3.4.2). Finally, reads were counted to genomic features using subreads (v.1.5.1) and GRCh37.87 (gene expression) or GRCh37.p13 (lncRNA expression).

mRNA-seq data of TCGA and RAQA datasets were normalized within lanes to correct for gene-specific effects (including GC-content and gene length) and between lanes to correct for sample-related differences (including sequencing depth) using the R package EDASeq (v.2.12.0). The resulting gene and lncRNA expression matrices were quantile normalized using R package preprocessCore (v.1.36.0). All downstream analysis was performed using R (v.3.5.1 or later).

### ICR consensus clustering

Consensus clustering of TCGA-BRCA samples was performed based on the expression values of 20 ICR genes using the ConsensusClusterPlus (v.1.42.0) and the following parameters: 5000 repeats, agglomerative hierarchical clustering with ward criterion inner and complete outer linkage. The optimal number of clusters for best segregation of samples was determined using the Calinski-Harabasz criterion, and samples were clustered as ICR high (immune hot), ICR medium or ICR low (immune cold). Downstream comparative analyses were performed using ICR high and ICR low tumor samples.

### ICR-differentially expressed lncRNA and protein-coding gene network analysis

Using the TCGA-BRCA dataset, we developed an analysis pipeline involving the identification of differentially enriched lncRNAs by ICR cluster and the construction of proxy protein-coding gene networks. Linear Model for Microarray Analysis (LIMMA, FDR p < 0.05) was applied to identify differentially expressed ir-lncRNAs between ICR high (n = 115) and ICR low (n = 128) tumors. Next, the differentially expressed ir-lncRNAs were mapped to a coding-non-coding gene (CNC) correlation network (Additional file 1A) as described in the LncRNAs2Pathways method [32]. The CNC network consists of 11,391 lncRNAs and 17,222 protein-coding genes. We utilized the random walk with restart (RWR) global network propagation algorithm to identify protein-coding genes that are most likely influenced by the ir-lncRNAs due to close proximity. Proximal protein-coding genes were identified based on their propagation scores as per the RWR algorithm.

### Pathway enrichment analysis

Once we defined the proximal coding genes associated with the differentially expressed ir-lncRNAs, we sought to explore their biological relevance through pathway enrichment analysis. First, we applied the approach described in the LncRNAs2Pathways method whereby a pathway enrichment score is calculated using the walkscores of the ranked protein coding genes in a Kolmogorov–Smirnov-like statistic with 1000 permutations. However, we observed that using this approach the walkscore distribution was highly skewed (Additional file 1B), whereby the majority of protein-coding genes have a very small walkscore (~ 0) and only a small fraction had a relatively high walkscore, which may result in false positive enriched pathways. Pathways consisting of predominantly protein-coding genes with small walkscores and few protein-coding genes with relatively high walkscore would be associated with smaller enrichment scores than pathways that were represented by protein-coding genes with primarily high walkscores. To address this limitation, we used a stringent criterion of a walkscore of ≥ 0.01 to generate a ranked list of most proximal protein-coding genes to the differentially expressed ir-lncRNAs, which coincidentally corresponds to approximately 1% of protein-coding genes. Next, we subjected the ranked protein-coding gene list to ConsensusPathDB [33, 34] and visualized the data by the func2vis R package (v.1.0.1) to identify the enriched pathways, and to Ingenuity Pathway Analysis (IPA) to identify enriched diseases and functions.

### Single-sample gene set enrichment analysis (ssGSEA)

Single sample gene set enrichment analysis was applied to calculate enrichment scores of specific gene sets within each individual sample using the GSVA R package (v.1.30.0).

### Correlation analysis

Spearman’s correlation analysis was used to assess the correlation between differentially expressed ir-lncRNAs and immune checkpoints. Spearman’s rank correlation coefficients were visualized in a heatmap using the ComplexHeatmap R package (v.2.1.2) with the columns ordered by sum of the correlation scores and the rows ordered by absolute sums of the correlation scores.

### Survival analysis

Univariate Cox proportional hazards regression survival analysis was performed using the survival R package (v.2.41–3), Hazard Ratios (HRs) between any two groups of interest and corresponding p values based on X2 test, and 95% confidence intervals (95%-CI) were calculated. Survival analysis was performed with the lncRNA signatures and ICR score as continuous variables and visualized in forest plots that were generated with the forestplot R package (v.1.7.2). The horizontal lines in the forest plot represent the 95% confidence intervals and the squares represent the Hazard ratios. In addition, univariate survival analysis was used to calculate the HRs of an ICR/3 ir-lncRNA combination model that sums the scaled enrichment scores of the ICR and 3 ir-lncRNA signatures. The Kaplan–Meier curves were generated using the ‘ggsurv’ function from survminer (v. 0.4.8) and the optimal cut-off point for stratification within each cancer type was determined by 5-fold cross validation analysis. Log-rank test was used to assess statistical differences in overall survival.

### Multivariate cox regression analysis

Multivariate Cox regression analysis was used to determine the contribution of individual lncRNAs to the prognostic value of the 3 lncRNA-signature using the survival package (v3.2-13).

### Akaike information criterion (AIC)

To determine whether the ICR or 3 ir-lncRNA signature is most likely to be the best model, we estimated and compared the Akaike information criterion (AIC) values using ‘extractAIC’ function from the stats package (v3.6.2).

### Cell composition deconvolution methods

We applied different deconvolution approaches to estimate the abundance of specific cell subsets from bulk transcriptomic data, including the Consensus Tumor MicroEnvironment cell estimation (Consensus^TME^) method [35] using ConsensusTME (v. 0.0.1.9), and immune cell subpopulation estimation methods based on leukocyte subgroup enrichment scores [36] or immune metagene expression profiling [37]. In addition, we applied the Estimation of STromal and Immune cells in MAlignant Tumor tissues using Expression data (ESTIMATE) algorithm [38] using ESTIMATE (v.1.0.13) to infer the extent of stromal and immune cell infiltration. Pearson scatter plots of the model enrichment scores with the 3 ir-lncRNA enrichment scores were generated using the corrplot (v. 0.92).

## Results

### LncRNA to coding-gene network analysis workflow

**Fig. 1:**
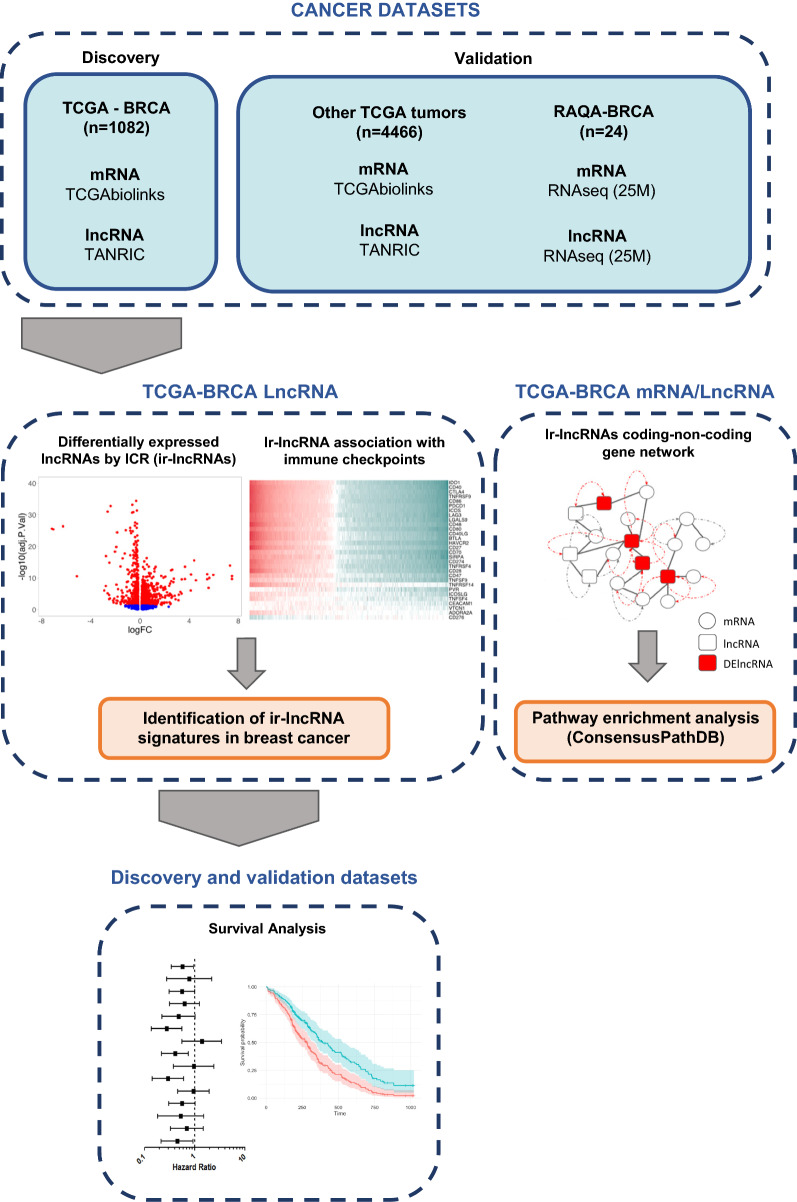
Visual representation of study workflow. We developed a multi-step pipeline to identify immune-related lncRNA signatures with prognostic connotation in solid cancers and their proxy protein-coding gene networks and signaling pathways

Few immune-related lncRNA signatures have been reported in cancer; however, their biological and clinical relevance and impact on downstream signaling pathways remain largely unexplored. To address this gap, we developed an analysis pipeline that involves the mapping of immune-related lncRNAs to coding-non-coding gene networks, followed by downstream analysis. The analysis pipeline was first applied to the TCGA breast cancer dataset whereby key findings were validated in other TCGA cancer datasets. We opted to use the TCGA breast cancer dataset as a discovery cohort given its large sample size, detailed clinical annotation, and robust prognostic significance of the ICR gene signature. First, we applied a 2-step process to the TCGA breast cancer dataset by identifying differentially expressed lncRNAs in immune favorable versus unfavorable breast tumors, followed by determining their proximal coding genes and their likely downstream biological effects through pathways and correlation analyses (Fig. 1). Finally, the prognostic value of lncRNA signatures was explored across multiple TCGA cancer datasets in addition to a smaller breast cancer cohort from Qatar.

### Identification of differentially expressed ir-lncRNAs by ICR tumor immune phenotype

**Fig. 2:**
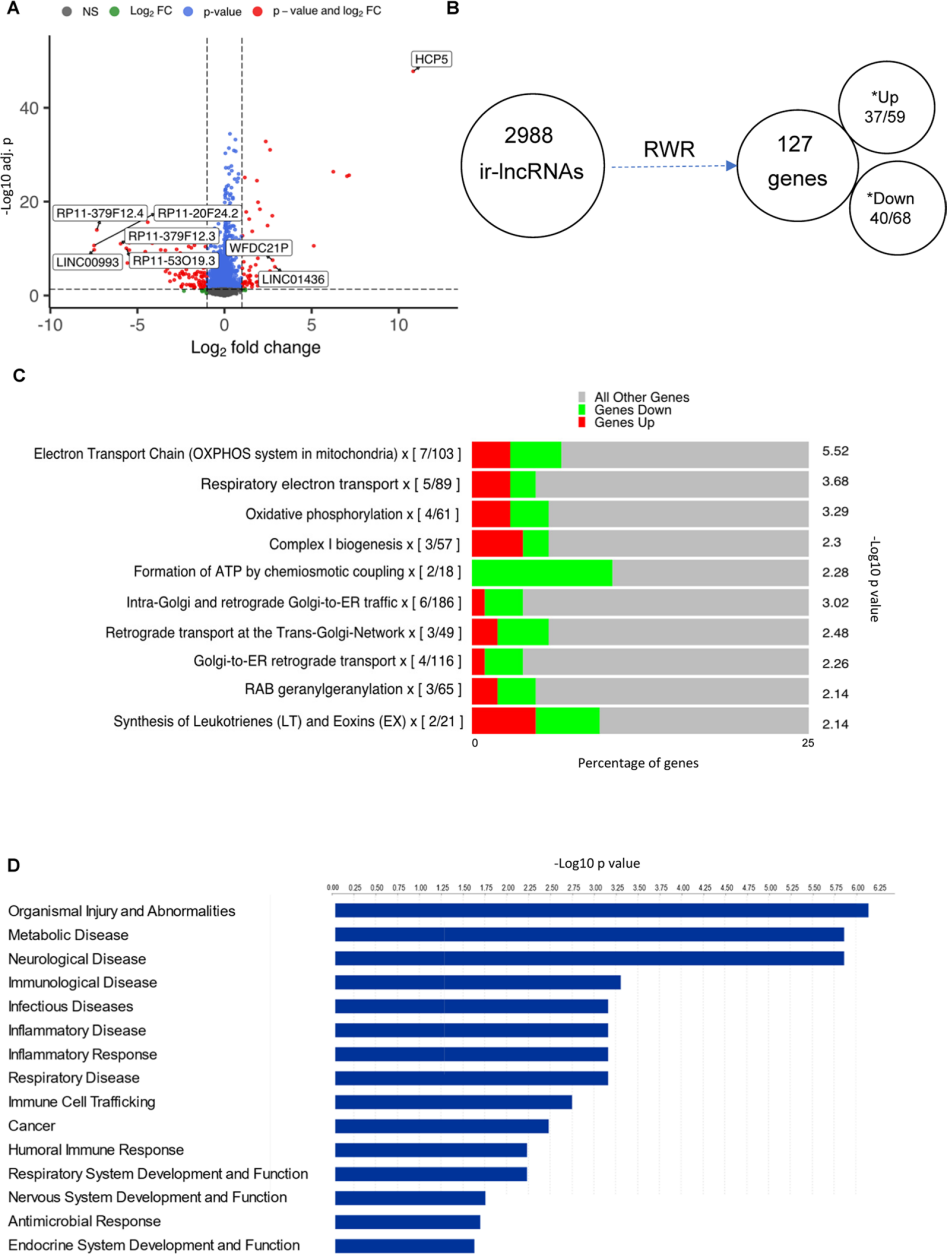
Breast cancer ICR-associated lncRNAs and associated biological pathways and functions. **A** Volcano plot of differentially expressed lncRNAs in ICR high vs low breast tumors from the TCGA breast cancer cohort. red = log2 fold change > 1 and adj p-value < 0.05, blue = adj p-value < 0.05, and green = adj p-value > 0.05. **B** Representation of the number of differentially expressed lncRNAs by ICR in TCGA-BRCA and their proximal protein-coding genes as per the RWR propagation algorithm. Protein coding genes with confirmed up- or downregulation as per differential expression analysis of RNAseq data are indicated with asterisks. The mRNA expression of 59 up- and 68 downregulated genes was available in the RNAseq data, and the differential expression of 37 and 40 genes was confirmed. **C** Pathway enrichment analysis of proxy protein-coding genes using ConsensusPathDB. For each pathway, the number of differentially expressed genes is indicated and the proportion of up- and down regulated genes in ICR high vs ICR low is visualized in red and green respectively. **D** IPA analysis of diseases and functions associated with top-ranked proxy protein-coding genes

TCGA breast tumor samples were classified into 3 subgroups based on the 20-gene ICR signature [27, 28, 39], and differentially expressed lncRNAs between ICR low (immune unfavorable) and ICR high (immune favorable) tumors were identified and labeled as immune related lncRNAs (ir-lncRNAs). Out of a total of 12,727 lncRNAs, we identified 2988 to be differentially expressed (FDR p < 0.05, log2FC > 1) of which 1284 were up- and 1704 were down-regulated in ICR high tumors (Fig. 2A). The top 5 ir-lncRNAs with the highest significant upregulation were HCP5, CTA-384D8.35, CTA-384D8.34, AC096579.7, CTA-384D8.31 and the top 5 significantly downregulated ir-lncRNAs included RP11-20F24.2, LINC00993, RP11-379F12.4, RP11-379F12.3 and RP11-53O19.3 (Additional file 2).

### Mapping of ir-lncRNA to proxy coding gene networks

Next, we applied the RWR global network propagation algorithm to map the 2988 ir-lncRNAs to the CNC network and computed propagation scores to identify protein-coding genes within the network that are most likely influenced by the ir-lncRNAs. Based on the propagation scores, a ranked list of 127 unique protein-coding genes with walkscores  ≥ 0.01 was compiled (Additional file 3). We then performed limma analysis on ICR high vs low breast tumors and found that out of the 127 predicted protein-coding genes, 37 and 40 were significantly up- and downregulated (FDR p < 0.05) respectively (Fig. 2B).

### Biological annotation of protein-coding gene networks indicates involvement in immune and metabolism pathways

To gain insight into the putative downstream biological roles of the differentially expressed ir-lncRNAs, we explored enriched pathways, diseases and functions associated with the 127 protein-coding genes. Pathway enrichment analysis revealed that pathways involved in ‘Electron Transport Chain (OXPHOS system in mitochondria)’, ‘Respiratory electron transport’, ‘Oxidative phosphorylation’, ‘Complex I biogenesis’ and ‘Formation of ATP by chemiosmotic coupling’ were the most significantly enriched. The first three pathways were mainly influenced by the differential expression of MT-ND1, MT-ND2, MT-CYB, NDUFB4, COX4I1, MT-ATP8 and MT-ATP6 genes (Fig. 2C, Additional file 4). Disease and function analysis identified several immunology related diseases and processes to be highly enriched in association with the 127 ir-lncRNA proxy protein-coding genes (p < 0.05), such as ‘Immunological disease’, ‘Infectious diseases’, ‘Inflammatory disease’, ‘Inflammatory response’, ‘Immune cell trafficking’, ‘Humoral immune response’ and ‘Antimicrobial response’ in addition to ‘Cancer’ (Fig. 2D, Additional file 4).

### Identification of ir-lncRNAs that are associated with the expression of multiple immune checkpoints

**Fig. 3:**
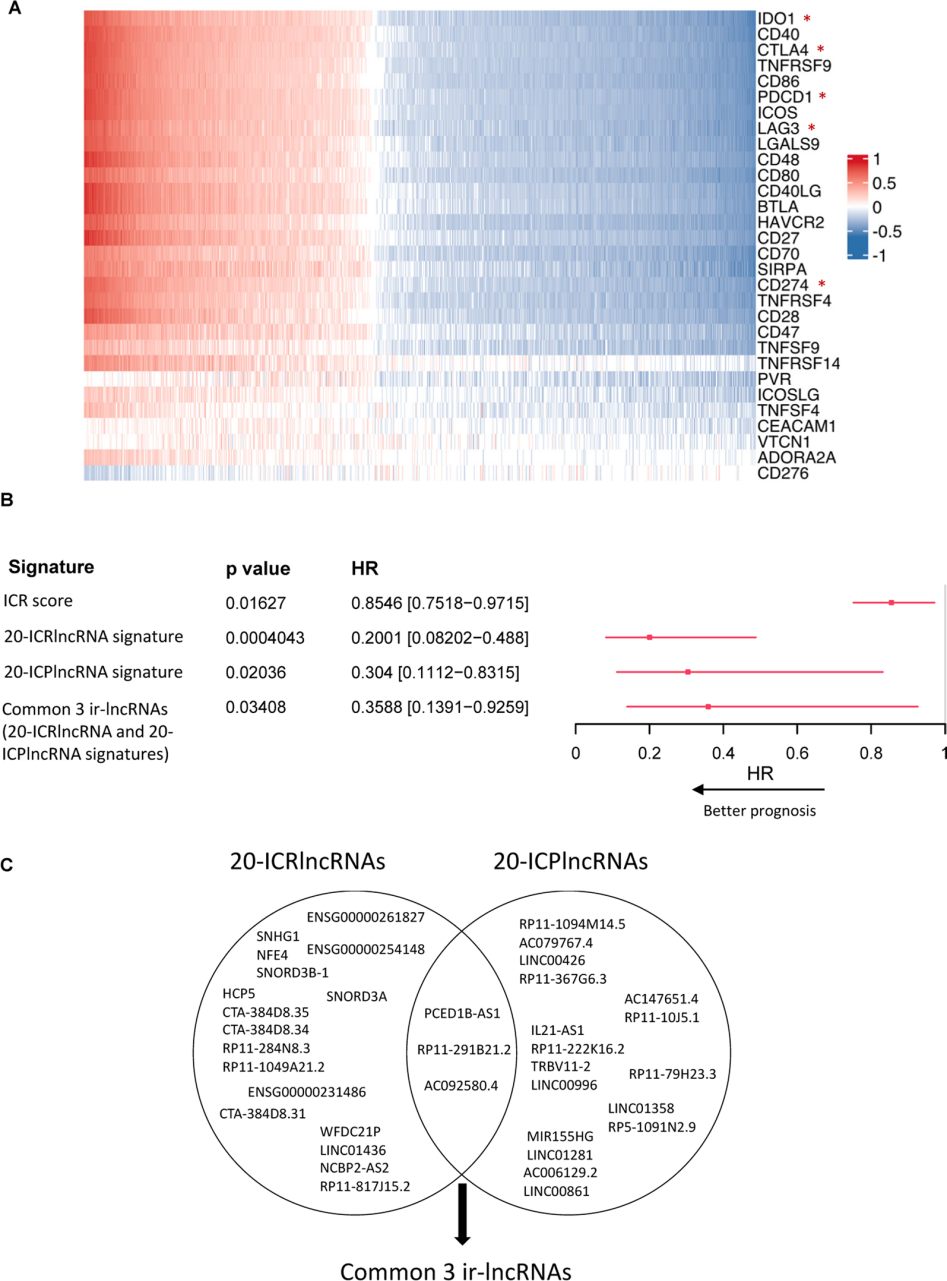
Comparison of ICR, 20-ICRlncRNA and 20-ICPlncRNA signature in TCGA breast cancer patients. **A** Heatmap of Spearman’s rank correlation coefficients between the expression of ir-lncRNAs and 30 immune checkpoints, color-coded from − 1 (dark blue) to +1 (dark red). Columns are ordered by the sum of the correlation scores and rows are ordered by the absolute sums of the correlation scores. Immune checkpoints that are included in the ICR signature are indicated with a red asterisk. **B** Forest plot showing HRs for death (overall survival) and corresponding 95%-confidence interval for single sample gene set enrichment scores of different immune signatures in the TCGA breast cancer cohort. Significant negative HRs are visualized in red. **C** The intersection of lncRNAs in the 20-ICRlncRNA and 20-ICPlncRNA signature in TCGA-BRCA. The three lncRNAs PCED1B-AS1, RP11-291B21.2 and AC092580.4 are represented in both signatures

In addition to its prognostic value, the ICR classifier has been suggested to potentially hold value as a predictor of response to immune checkpoint blockade [27, 40]. Furthermore, several lncRNAs have been found to be involved in the regulation of the immune checkpoint expression [41]. Hence, we further explored the correlation of the differentially expressed ir-lncRNAs with immune checkpoints in the TCGA-BRCA cohort using Spearman correlation analysis (Fig. 3A, Additional file 5). Overall, similar correlation patterns were observed between individual ir-lncRNAs and immune checkpoints. Notably, CD276 (B7-H3) was the only immune checkpoint that showed an opposite correlation pattern with ir-lncRNA expression.

### Ir-lncRNA signatures with prognostic value in breast cancer

Next, we investigated the prognostic value of two ir-lncRNA signatures in breast cancer, the first consisting of the top 20 differentially expressed ir-lncRNAs by ICR (20-ICRlncRNA) and the second of the top 20 differentially expressed ir-lncRNAs associated with immune checkpoints (20-ICPlncRNA). Both ir-lncRNA signatures conferred a significant survival benefit (20-ICRlncRNA HR = 0.2001 [95% CI 0.08202–0.488], p < 0.001; 20-ICPlncRNA HR = 0.304 [95% CI 0.1112–0.8315], p = 0.02036), with the 20-ICRlncRNA signature even outperforming the ICR score itself (HR = 0.8546 [95% CI 0.7518–0.9715], p = 0.01627) (Fig. 3B). Interestingly, the two ir-lncRNA signatures shared 3 common ir-lncRNAs (PCED1B-AS1 (ENSG00000247774), RP11-291B21.2 (ENSG00000256039) and AC092580.4 (ENSG00000235576), Fig. 3C) which together constitute a much smaller signature that retains prognostic significance (HR = 0.3588 [95% CI 0.1391–0.9259], p = 0.03408) in a more practical format for clinical use.

### Ir-lncRNA signatures demonstrate prognostic significance across multiple tumor types

**Fig. 4:**
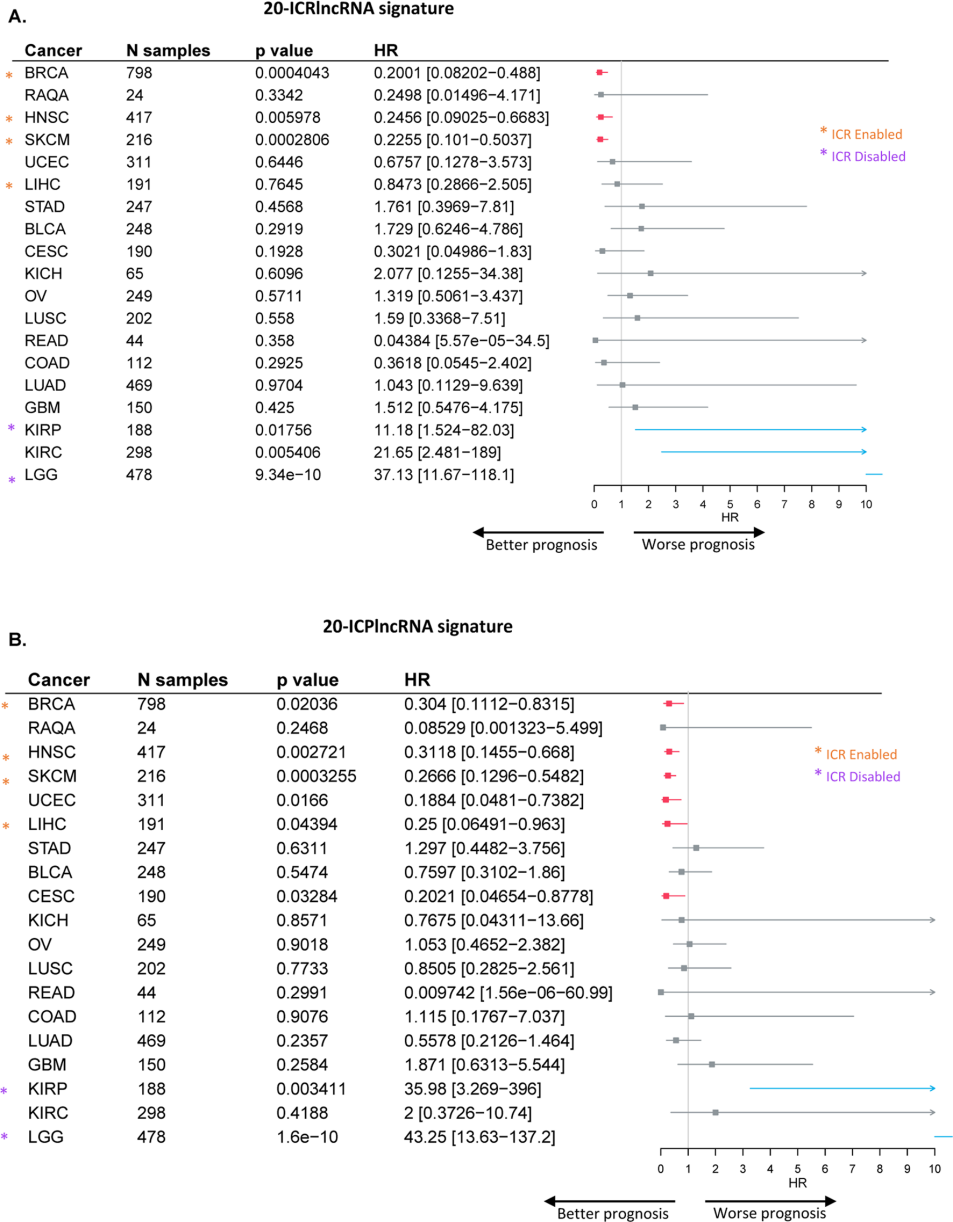
Prognostic significance of 20-ICRlncRNA and 20-ICPlnRNA signature in solid cancers. Forest plot showing HRs for death (overall survival) and corresponding 95%-confidence interval for single sample gene set enrichment scores of the **A** 20-ICRlncRNA and **B** 20-ICPlncRNA signature, p-values, and the number of patients for each TCGA cancer cohort and RAQA breast cancer cohort. Significant positive HRs are visualized in blue and significant negative HRs are visualized in red. ICR enabled (HR < 1, p-value < 0.05) cancer types are indicated with orange asterisks and ICR disabled (HR > 1, p-value < 0.05) cancers are indicated with purple asterisks

Given the prognostic connotation of the ir-lncRNA signatures in breast tumors, we sought to assess its clinical value across different solid tumor types in comparison with the ICR classifier. For this purpose, we included an additional 17 TCGA datasets for which both gene and lncRNA expression data are available as well as one small breast cancer dataset from Qatar (RAQA). Forest plot results (Fig. 4) show that both 20-lncRNA signatures are significantly associated with better overall survival in head and neck squamous cell carcinoma (HNSC, 20-ICRlncRNA HR = 0.2456 [95% CI 0.09025–0.6683] and 20-ICPlncRNA HR = 0.3118 [95% CI 0.1455–0.668], p < 0.01) and skin cutaneous melanoma (SKCM, 20-ICRlncRNA HR = 0.2255 [95% CI 0.101–0.5037] and 20-ICPlncRNA HR = 0.2666 [95% CI 0.1296–0.5482], p < 0.001) in addition to breast cancer (BRCA), whereas the opposite was true in kidney renal papillary cell carcinoma (KIRP, 20-ICRlncRNA HR = 11.18 [95% CI 1.524–82.03], p = 0.01765, 20-ICPlncRNA HR = 35.98 [95% CI 3.269–396], p < 0.01) and low-grade glioma (LGG, 20-ICRlncRNA HR = 37.13 [95% CI 11.67–118.1] and 20-ICPlncRNA HR = 43.25 [95% CI 13.63–137.2], p < 0.001). Furthermore, the 20-ICRlncRNA signature (Fig. 4A) was negatively correlated with overall survival in kidney renal clear cell carcinoma (KIRC, HR = 21.65 [95% CI 2.481–189], p < 0.01), while the 20-ICPlncRNA signature (Fig. 4B) was positively correlated with survival in uterine corpus endometrial carcinoma (UCEC, HR = 0.1884 [95% CI 0.0481–0.7382], p = 0.0166), liver hepatocellular carcinoma (LIHC, HR = 0.25 [95% CI 0.06491–0.963], p = 0.04394) and cervical squamous cell carcinoma and endocervical adenocarcinoma (CESC, HR = 0.2021 [95% CI 0.04654–0.8778], p = 0.03284). In accordance with our previous work [28], we classified each tumor cohort with available lncRNA data as either ‘ICR enabled’ (HR < 1 with a p-value < 0.05), ‘ICR disabled’ (HR > 1 with a p-value < 0.05), or ‘ICR neutral’ (p-value > 0.05) as based on the prognostic connotation of the ICR score (Additional file 6). Interestingly, all ICR-enabled tumors (BRCA, HNSC, SKCM, LIHC) were associated with a favorable prognostic ir-lncRNA signature and conversely, all ICR disabled tumors (KIRP, LGG) were characterized by an unfavorable prognostic ir-lncRNA signature (Fig. 4).

### Performance of 3 Ir-lncRNA signature as a prognostic classifier in cancer

**Fig. 5:**
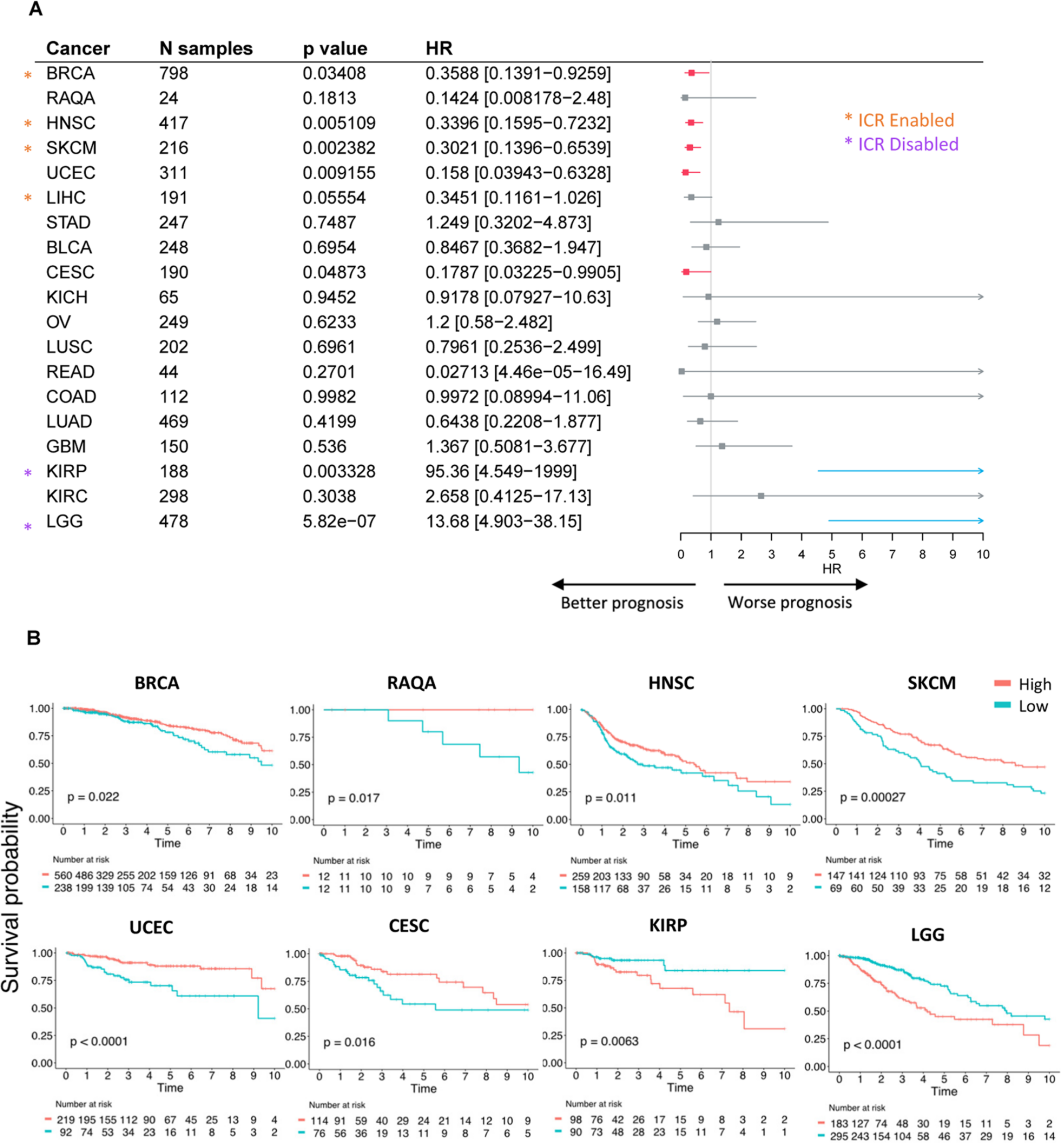
Prognostic significance of 3 ir-lncRNAs signature in solid cancers. **A** Forest plot showing HRs for death (overall survival) and corresponding 95%-confidence interval for single sample gene set enrichment scores of the 3 ir-lncRNA signature and the number of patients for each TCGA cancer cohort and RAQA breast cancer cohort. Significant positive HRs are visualized in blue and significant negative HRs are visualized in red. ICR enabled (HR < 1, p-value < 0.05) cancer types are indicated with orange asterisks and ICR disabled (HR > 1, p-value < 0.05) cancers are indicated with purple asterisks. **B** Overall survival Kaplan–Meier curves of selected cancers, dichotomized by the enrichment score of the 3 ir-lncRNAs signature. Dichotomization cutoff of ‘high’ (red) and ‘low’ (cyan) subgroups was based on optimal cut-off points as determined by fivefold cross validation analysis. Cancer types in which the 3 ir-lncRNA signature holds significant prognostic value according to the logrank test were selected for visualization. Censor points are indicated by vertical lines

Similarly, we assessed the prognostic value of the 3 ir-lncRNA signature across all 18 solid tumor types (Fig. 5A). In analogy with the ICR signature, we found that the 3 ir-lncRNA signature was associated with better prognosis in breast cancer (BRCA, HR = 0.3588 [95% CI 0.1391–0.9259], p = 0.03408), head and neck squamous cell carcinoma (HNSC, HR = 0.3396 [95% CI 0.1595–0.7232], p < 0.01) and skin cutaneous melanoma (SKCM, HR = 0.3021 [95% CI 0.1396–0.6539], p < 0.01), while demonstrating a negative association with survival in kidney renal papillary cell carcinoma (KIRP, HR = 95.36 [95% CI 4.549–1999], p < 0.01) and low-grade glioma (LGG, HR = 13.68 [95% CI 4.903–38.15], p < 0.001). Furthermore, the 3 ir-lncRNA signature, but not the ICR signature, held prognostic significance in uterine corpus endometrial carcinoma (UCEC, HR = 0.158 [95% CI 0.03943–0.6328], p < 0.01) and cervical squamous cell carcinoma and endocervical adenocarcinoma (CESC, HR = 0.1787 [95% CI 0.03225–0.9905], p = 0.04873). Although no significant association was found in the Qatari breast cancer cohort, most likely to the small sample size, a clear trend for better survival was observed (HR = 0.1424 [95% CI 0.008178–2.48], ns). The prognostic performance of the 3 ir-lncRNA signature as determined by the Akaike information criterion (AIC) demonstrated a similar performance as the ICR classifier across solid cancers (Additional file 7), with the exception of uterine corpus endometrial carcinoma where the 3 ir-lncRNA signature was the better model (UCEC, dAIC = − 4.3) and skin cutaneous melanoma where the ICR signature was found to be the best model (SKCM, dAIC = 6.0). Given the smaller size of the 3 ir-lncRNA signature, it provides a better ease-of-use for clinical practice even in cancer types where both models perform equally well. In addition, 5-fold cross-validated Kaplan Meier survival curves with log-rank test (Fig. 5B, Additional file 8) corroborated the prognostic value of the 3 ir-lncRNA signature in breast cancer, head and neck squamous cell carcinoma and skin cutaneous melanoma (BRCA, HNSC, SKCM; ICR enabled tumor types), uterine corpus endometrial carcinoma and cervical squamous cell carcinoma and endocervical adenocarcinoma (UCEC, CESC; ICR neutral tumor types), and kidney renal papillary cell carcinoma and low-grade glioma (KIRP, LGG; ICR disabled tumor types). Survival analysis of the RAQA breast cancer cohort showed a clear bifurcation of overall survival (p = 0.017) despite the smaller size of the cohort. Due to limitations with cohort size and event numbers, we were not able to perform the 5-fold cross-validation analyses on the rectum adenocarcinoma (READ) and colon adenocarcinoma (COAD) datasets. Multivariate Cox regression analysis of the three individual ir-lncRNAs (Table 1) showed that out of the three lncRNAs, RP11-291B21.2 was most often associated with survival (STAD, GBM, KIRP, KIRC), followed by AC092580.4 (BRCA, STAD, LGG) and PCED1B-AS1 (LUAD, KIRP).

**Table 1:** Multivariate analysis of the enrichment scores of the 3 ir-lncRNAs in cancer

Cancer	Gene name	HR	Cox p value, multivariate	CI lower	CI upper	log rank p, multivariate	Cox p value 3, lncRNAs signature
BRCA	AC092580.4	0.59	0.03	0.37	0.94	0.02	0.03
	PCED1B-AS1	1.01	0.90	0.85	1.20		
	RP11-291B21.2	0.98	0.90	0.65	1.46		
HNSC	AC092580.4	0.98	0.78	0.88	1.10	0.02	2.38E-03
	PCED1B-AS1	0.86	0.23	0.68	1.10		
	RP11-291B21.2	0.88	0.18	0.72	1.06		
RAQA	AC092580.4	3.66E-06	1.00	0.00	Inf	0.50	0.14
	PCED1B-AS1	1.00	0.97	0.79	1.28		
	RP11-291B21.2	1.28E-03	1.00	0.00	Inf		
SKCM	AC092580.4	0.87	0.19	0.70	1.07	0.04	2.38E-03
	PCED1B-AS1	0.98	0.71	0.86	1.11		
	RP11-291B21.2	0.97	0.81	0.70	1.27		
UCEC	AC092580.4	–	–	–	–	0.39	0.01
	PCED1B-AS1	0.88	0.39	0.66	1.18		
	RP11-291B21.2	–	–	–	–		
LIHC	AC092580.4	0.92	0.55	0.70	1.20	0.61	0.06
	PCED1B-AS1	1.14	0.46	0.80	1.63		
	RP11-291B21.2	0.57	0.33	0.18	1.76		
STAD	AC092580.4	0.73	0.01	0.57	0.93	0.09	0.75
	PCED1B-AS1	1.02	0.84	0.80	1.30		
	RP11-291B21.2	1.36	0.05	1.00	1.85		
BLCA	AC092580.4	1.05	0.40	0.93	1.19	0.43	0.07
	PCED1B-AS1	0.98	0.70	0.89	1.09		
	RP11-291B21.2	0.87	0.10	0.70	1.05		
CESC	AC092580.4	0.95	0.60	0.79	1.15	0.06	0.05
	PCED1B-AS1	0.66	0.20	0.35	1.25		
	RP11-291B21.2	1.02	0.80	0.83	1.27		
KICH	AC092580.4	0.01	0.12	1.18E-05	3.91	2.81E-07	0.95
	PCED1B-AS1	3.50	0.16	0.62	20.12		
	RP11-291B21.2	877.00	0.51	1.49E-06	5.17E + 11		
OV	AC092580.4	1.07	0.74	0.70	1.62	0.90	0.62
	PCED1B-AS1	0.82	0.44	0.49	1.37		
	RP11-291B21.2	1.03	0.97	0.30	3.50		
LUSC	AC092580.4	0.96	0.71	0.77	1.20	0.50	0.70
	PCED1B-AS1	1.08	0.46	0.88	1.33		
	RP11-291B21.2	0.89	0.41	0.66	1.19		
READ	AC092580.4	–	–	–	–	0.19	0.27
	PCED1B-AS1	0.01	0.24	8.05E-07	35.58		
	RP11-291B21.2	–	–	–	–		
COAD	AC092580.4	–	–	–	–	0.96	1.00
	PCED1B-AS1	0.98	0.96	0.35	2.69		
	RP11-291B21.2	–	–	–	–		
LUAD	AC092580.4	1.00	0.98	0.90	1.12	0.20	0.42
	PCED1B-AS1	0.88	0.05	0.77	1.00		
	RP11-291B21.2	1.04	0.46	0.93	1.17		
GBM	AC092580.4	0.97	0.86	0.72	1.32	0.10	0.54
	PCED1B-AS1	1.00	0.93	0.92	1.08		
	RP11-291B21.2	32.10	0.01	2.03	507.13		
KIRP	AC092580.4	1.37	0.18	0.86	2.20	1.30E-06	3.33E-03
	PCED1B-AS1	0.77	0.05	0.60	1.00		
	RP11-291B21.2	15.50	9.87E-05	3.90	61.58		
KIRC	AC092580.4	1.03	0.78	0.85	1.24	5.17E-10	0.30
	PCED1B-AS1	1.08	0.34	0.92	1.27		
	RP11-291B21.2	1.19	3.00E-03	1.06	1.33		
LGG	AC092580.4	6.90	1.64E-05	2.87	16.70	3.71E-08	5.82E-07
	PCED1B-AS1	0.99	0.84	0.86	1.13		
	RP11-291B21.2	1.28	0.62	0.48	3.40		

Next, we sought to investigate whether combining the enrichment scores of the 3 ir-lncRNA and ICR signatures may improve prognostic significance (Table 2). We found that the combined model performed equally well as the 3 ir-lncRNA or the ICR signature in many cancers which is to be expected as the signatures are strongly correlated. In some cases, the combined model performed less well than the individual ICR and 3 ir-lncRNA signatures. For instance, in uterine corpus endometrial carcinoma (UCEC), the 3 ir-lncRNA signature remained the strongest prognostic predictor (HR = 0.2662 [95% CI 0.09837–0.7202], p = 0.009155) compared to the ICR signature (HR = 0.8485 [95% CI 0.6783–1.061], p = 0.1505) or the combined model (HR = 0.8458 [95% CI 0.6958–1.028], p = 0.09279).

**Table 2:** Univariate analysis of ICR, 3 ir-lncRNA and combination ICR/3 ir-lncRNA model

Cancer	n	ICR signature		3 ir-lncRNA signature		ICR/3 ir-lncRNA signature	
		p-value	HR [CI]	p-value	HR [CI]	p-value	HR [CI]
BRCA	798	0.01617	0.8636 [0.7664–0.9732]	0.03408	0.4794 [0.2429–0.9463]	0.01525	0.8784 [0.7911–0.9754]
RAQA	24	0.4672	0.8761 [0.6134–1.251]	0.1813	0.2471 [0.03183–1.918]	0.3824	0.8583 [0.6091–1.209]
HNSC	417	0.007468	0.8955 [0.8259–0.9709]	0.00511	0.4609 [0.268–0.7926]	0.005836	0.9024 [0.8388–0.9707]
SKCM	216	0.000243	0.8287 [0.7496–0.9162]	0.002382	0.4238 [0.2435–0.7374]	0.000243	0.8496 [0.7787–0.9269]
UCEC	311	0.1505	0.8485 [0.6783–1.061]	0.009155	0.2662 [0.0984–0.7202]	0.09279	0.8458 [0.6958–1.028]
LIHC	191	0.0983	0.89 [0.7752–1.022]	0.05554	0.4662 [0.2134–1.018]	0.07708	0.8926 [0.7869–1.012]
STAD	247	0.2773	0.9391 [0.8385–1.052]	0.7487	1.173 [0.4419–3.114]	0.3354	0.9506 [0.8575–1.054]
BLCA	248	0.3766	0.9567 [0.8672–1.055]	0.6954	0.8875 [0.4884–1.613]	0.4031	0.964 [0.8845–1.051]
CESC	190	0.03583	0.8286 [0.6951–0.9876]	0.04873	0.2908 [0.08516–0.993]	0.03316	0.8413 [0.7177–0.9863]
KICH	65	0.5425	0.8991 [0.6385–1.266]	0.9452	0.9403 [0.1623–5.447]	0.5853	0.9214 [0.6868–1.236]
OV	249	0.6857	0.9801 [0.8893–1.08]	0.6233	1.14 [0.6766–1.919]	0.7909	0.9888 [0.9101–1.074]
LUSC	202	0.2156	0.9207 [0.8079–1.049]	0.6961	0.8491 [0.3738–1.929]	0.2505	0.9352 [0.8341–1.048]
READ	44	0.1527	0.3151 [0.06472–1.534]	0.2701	0.0752 [0.00076–7.467]	0.1497	0.3248 [0.07033–1.5]
COAD	112	0.9295	1.012 [0.7783–1.316]	0.9982	0.998 [0.1777–5.604]	0.9357	1.01 [0.7939–1.285]
LUAD	469	0.8737	0.993 [0.9108–1.083]	0.4199	0.7292 [0.3384–1.571]	0.8207	0.9908 [0.9148–1.073]
GBM	150	0.5058	1.043 [0.9216–1.18]	0.536	1.251 [0.6153–2.545]	0.4844	1.041 [0.9308–1.163]
KIRP	188	7.63E-05	1.511 [1.231–1.853]	0.003328	26.28 [2.964–233.1]	5.14E-05	1.477 [1.223–1.784]
KIRC	298	0.1702	1.094 [0.9621–1.245]	0.3038	2.016 [0.5298–7.672]	0.1689	1.088 [0.9648–1.227]
LGG	478	3.14E-09	1.37 [1.234–1.52]	5.82E-07	6.528 [3.128–13.63]	7.04E-10	1.341 [1.222–1.472]

### Association of 3 Ir-lncRNA signature with tumor features

**Fig. 6:**
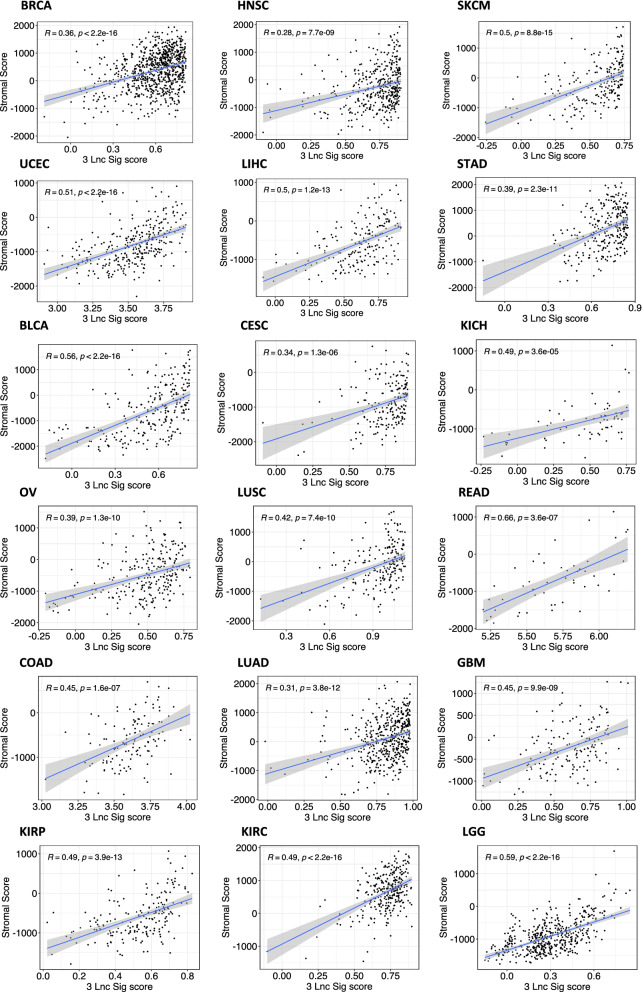
Correlation of ESTIMATE stromal scores with the 3 ir-lncRNA signature. Scatter plots showing the correlation between the ESTIMATE stromal scores and the 3 ir-lncRNA enrichment scores for each cancer type. R represents the Pearson correlation coefficient

**Fig. 7:**
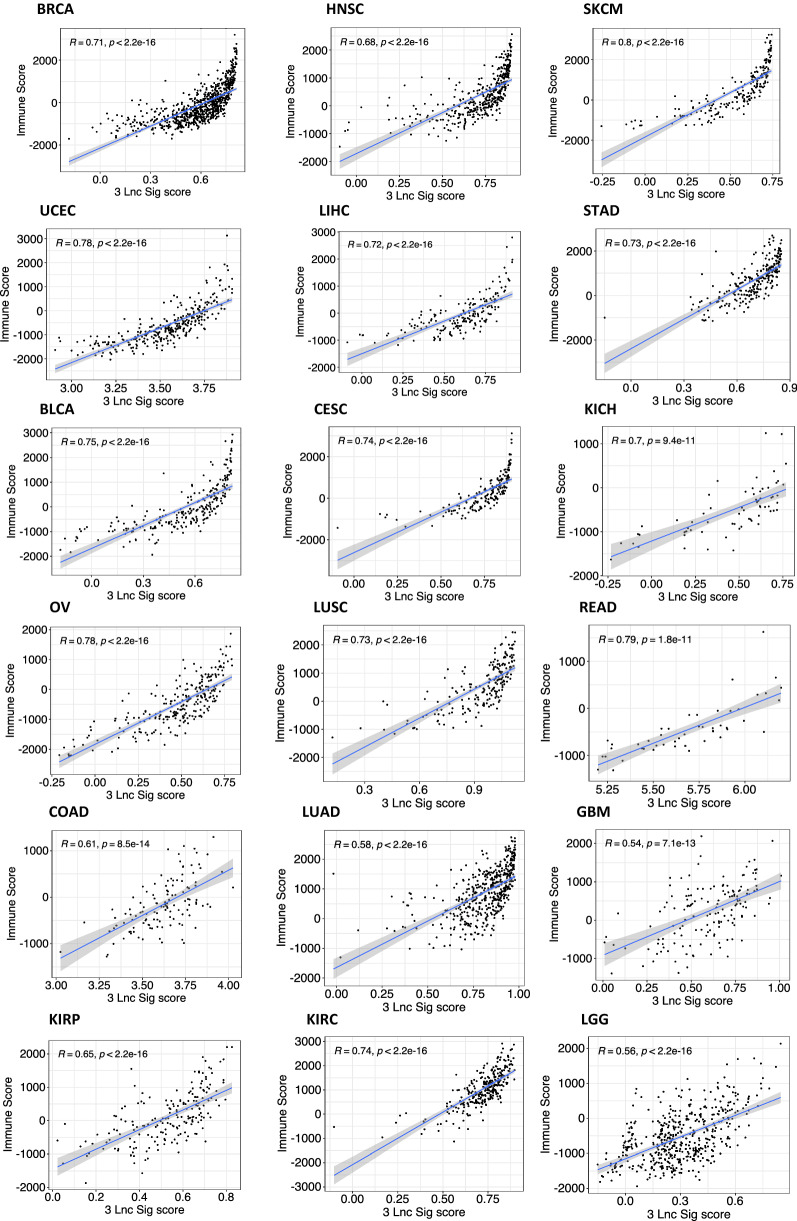
Correlation of the ESTIMATE immune scores with the 3 ir-lncRNA signature. Scatter plots showing the correlation between the ESTIMATE immune scores and the 3 ir-lncRNA enrichment scores for each cancer type. R represents the Pearson correlation coefficient

Finally, we sought to investigate whether the 3 ir-lncRNA signature shows any significant association with prognostic tumor features such as stromal cell composition and immune cell infiltration. Using the ESTIMATE algorithm, we calculated the stromal (Fig. 6) and immune scores for each cancer type and determined their correlation with the 3 ir-lncRNA enrichment score (Fig. 7). We found moderate to strong correlations between the 3 ir-lncRNA signature and the ESTIMATE stromal score in most cancer types with the strongest correlation in rectum adenocarcinoma (READ, R = 0.66, p < 0.001) and low-grade glioma (LGG, R = 0.59, p < 0.001). Not entirely unexpected we found moderate to very strong correlations between the 3 ir-lncRNA signature and the ESTIMATE immune score in all tumor types with the strongest associations found in skin cutaneous melanoma (SKCM, R = 0.8), rectum adenocarcinoma (READ, HR = 0.79), uterine Corpus Endometrial Carcinoma (UCEC, R = 0.78) and ovarian serous cystadenocarcinoma (OV, R = 0.78). These findings suggest that the expression of the 3 ir-lncRNAs in our signature likely originates from both the stromal and immune cell compartment. Furthermore, we used two distinct deconvolution methods to estimate the relative cell composition of the tumor microenvironment in association with the 3 ir-lncRNA signature; the first using leukocyte subgroup enrichment scores and the second being the Consensus^TME^ approach [35, 36] (Additional file 9). Correlation heatmaps revealed similar patterns across tumor types, showing an overall positive association of the 3 ir-lncRNA signature with pro-inflammatory and cytotoxic immune cells from the adaptive and innate immune system and an inverse association with Th2 helper cells, Th17 cells, T cell memory cells and immunomodulatory NK CD56bright.

## Discussion

The vast amount of tumor immunology research studies and immunotherapy clinical trials have clearly demonstrated the importance of the tumor immunophenotype in clinical outcome and highlighted the need for predictive biomarkers of an active tumor immune microenvironment. In our previous work, we defined and validated the ICR signature as a prognostic tool to distinguish ‘hot’ tumors (ICR high) from ‘cold’ tumors (ICR low), whereby, the former are associated with a more favorable clinical outcome and greater treatment response to immune checkpoint blockade [27, 28]. Mechanistically, we found that ICR low tumors are strongly associated with mutations in MAPK and activation of the TGF-β and Wnt-β catenin pathways [27, 42]. In a recent pan-cancer analysis, we further demonstrated that the prognostic connotation of the ICR immune phenotype may be differentially impacted by the activation of distinct oncogenic pathways [28]. As such, the favorable prognosis associated with ICR high tumors was abolished by the activation of TGF-β signaling and a low proliferation molecular profile.

In the present study, we expanded our molecular analysis of ICR immune phenotypes to include lncRNAs as potential regulators of immune disposition and concomitantly immunotherapy response. Analysis of the lncRNA profile of ICR high versus ICR low breast tumors from the TCGA repository revealed a number of differentially expressed immune-related lncRNAs (ir-lncRNAs) which we subsequently mapped to a coding-non-coding gene network using a computational network propagation algorithm. Pathway analysis of those proxy protein-coding genes subsequently identified the genes to be involved in multiple biological processes related to metabolic pathways and protein trafficking. Several of the identified processes play a major role in mitochondrial oxidative phosphorylation which largely defines the metabolic fitness of cancer and immune cells. Generally, as tumors progress cancer cells undergo metabolic reprogramming from oxidative phosphorylation to aerobic glycolysis in order to support growth and survival. This reprogramming creates an environment of metabolic competition for glucose between cancer cells and tumor infiltrating cytotoxic T cells who also increasingly rely on aerobic glycolysis upon activation [43, 44]. As such, metabolic competition can lead to T cell dysfunction, resulting in unfavorable tumor immune phenotypes. Furthermore, the synthesis of leukotrienes and eoxins plays an important role in shaping the tumor microenvironment by regulating leukocyte migration and promoting tumor growth and metastasis [45]. Together, this suggests that ir-lncRNAs may be implicated in defining the immune contexture of tumors in addition to promoting tumorigenesis. The presence of an active pre-existing immune response is a crucial determining factor in immunotherapy response, in particular to immune checkpoint blockade.

To address the role of ir-lncRNAs in immune checkpoint expression, we investigated the relationship of ir-lncRNA signatures with 30 immune checkpoint molecules in breast cancer. We found that ICR-associated ir-lncRNAs could be categorized into two clusters, one with positive and one with negative correlations with immune checkpoint molecules. In exception, CD276 (B7-H3) expression showed an opposite correlation with ir-lncRNA expression. CD276 is expressed in many cell types and has been shown to play a role in innate and adaptive immune responses, however, its function as a co-stimulatory or co-inhibitory molecule remains controversial [46].

Finally, we sought to determine the prognostic value of ir-lncRNAs based on our findings that showed an association of ir-lncRNAs with metabolic activities and immune checkpoint expression, which both regulate immune cell disposition and therefore may impact clinical outcome. We defined three different ir-lncRNA signatures using the TCGA breast cancer dataset, evaluated their prognostic significance in a local breast cancer cohort and explored their clinical value in a pan-cancer setting. Although the local breast cancer cohort (RAQA) is considerably small in size, similar patterns in prognostic significance were observed, highlighting the robustness of the ir-lncRNA signatures across ancestral populations such as the Arab population which remains largely underrepresented. The first signature comprised the top 20 differentially expressed ir-lncRNA in ICR high versus ICR low tumors (20-ICRlncRNA) and demonstrated prognostic significance in 6 solid tumor types (BRCA, HNSC, SKCM, KIRP, KIRC and LGG) with a lower hazard ratio for overall survival than the ICR signature. The second lncRNA signature is composed out of the top 20 ir-lncRNAs that are positively correlated with immune checkpoint expression (20-ICPlncRNA) and overall shows a stronger effect on survival than the ICR signature. Further study is needed to investigate the individual checkpoint molecule correlations with the 20-ICPlncRNA signature in order to gain insight into potential molecular mechanisms and to explore their value in predicting immunotherapy response in larger prospective cancer patient cohorts. Comparison of the two ir-lncRNA signatures revealed the presence of three common ir-lncRNAs, PCED1B-AS1, RP11-291B21.2 and AC092580.4, that could potentially be used as a minimal informative set of ir-lncRNAs with prognostic significance and more practical format for clinical use compared to the ICR signature. Survival analyses of the 3 ir-lncRNA signature confirmed its prognostic value in 7 cancer types; 5 in which it showed a stronger effect on survival compared to the ICR signature (ICR enabled [BRCA, HNSC, SKCM], ICR disabled [KIRP, LGG]) and 2 in which the ICR does not hold prognostic significance (ICR neutral [UCEC, CESC]). These findings suggest that the 3 ir-lncRNA signature could be used to improve prognostic stratification over the ICR and in addition could offer prognostic information in tumors where ICR does not hold prognostic value (ICR neutral). Of note, whereas both signatures show a positive correlation with overall survival in the majority of cancer types, they are associated with a worse survival in kidney renal papillary cell carcinoma (KIRP) and low-grade glioma (LGG). In accordance, several studies have reported an inverse association between high immune cell infiltration or immune activity with prognosis in these specific tumor types. For instance, in low-grade glioma a worse prognosis has been associated with enhanced immune infiltration whereby an increase in M0/M1 macrophages increases the permeability of the blood brain barrier and promotes glioma cell growth and invasion [47–49]. In addition, high B cell infiltration has been associated with worse prognosis in kidney renal papillary cell carcinoma (KIRP) and low-grade glioma (LGG) and may be linked to the presence of a specific immunosuppressive B cell subset, regulatory B cells [50, 51]. Moreover, despite the presence of tumor immune cell infiltration, T cell function may be suppressed by an increase in immune checkpoint expression as has been suggested by a 15-gene signature in kidney renal papillary cell carcinoma (KIRP) [52]. Further study is needed to tease out the relation between KIRP and LGG patient survival and the abundance and functionality of diverse immune cell subsets.

In order to further investigate the association of the 3 lncRNAs with multiple cell types within the tumor microenvironment, we used several deconvolution methods. Using the ESTIMATE algorithm, we found that the 3 ir-lncRNA signature strongly correlates with both the ESTIMATE stromal and immune scores across cancers, suggesting that the 3 ir-lncRNA expression might be derived from both the stromal and immune cell compartment within the tumors. Furthermore, we found an overall positive association of the 3 ir-lncRNA signature with pro-inflammatory and cytotoxic immune cell subpopulations, and a negative correlation with T helper 2 and T helper 17 cells, T cell memory cells and immunomodulatory NK CD56bright cells.

Given the potential clinical value of the 3 ir-lncRNA signature, we looked into the reported molecular mechanisms and biological processes that may be affected by these 3 ir-lncRNAs. All three ir-lncRNAs have been found to be overexpressed in multiple cancer types [53–61]. Mechanistically, PCED1B-AS1 has been shown to function as an oncogenic lncRNA regulating miRNA expression, ultimately promoting aerobic glycolysis, proliferation, invasion and epithelial-to-mesenchymal transition while reducing apoptosis of cancer cells [53, 56, 57, 59]. In addition, PCED1B-AS1 was found to be positively associated with immune checkpoint expression and in particular to increase the expression of PD-L1 and PD-L2 through interaction with mir-194-5p, leading to an enhanced immunosuppression [10, 58]. Less is known about the function of RP11-291B21.2 in cancer, however, it has been associated with durvalumab response in non-small cell lung cancer and bladder cancer patients, and was found to correlate with several key immune genes [62]. Single-cell RNAseq analysis further indicates that RP11-291B21.2 is dominantly expressed in exhausted CD8+ T cells [62]. Furthermore, AC092580.4 expression is strongly correlated with key immune genes and pathways including Gata3 expression, suggesting that it may be involved in modulating T cell polarization and hence anti-tumor immunity [63, 64]. Additional single cell multi-omics and functional studies are needed to better characterize the cellular origin and interacting partners and downstream signaling pathways of each of these ir-lncRNAs.

## Conclusions

In summary, our findings indicate that the 3 ir-lncRNA signature holds prognostic value in multiple solid cancer types with stronger effects on overall survival than the well-established 20-gene ICR signature, in particular in uterine corpus endometrial carcinoma. Moreover, given the smaller size of the lncRNA signature it provides a greater ease-of-use for clinical implementation, warranting the need for larger, prospective studies to validate its clinical utility.

## Supplementary Information

**Additional file 1**: Mapping of differentially expressed ir-lncRNAs to protein coding genes. (A) Diagram representation of the random walk with restart global propagation network algorithm. (B) Walkscore distribution of protein-coding genes in TCGA-BRCA, with cutoff set at walkscore ≥ 0.01 to generate a ranked list of protein-coding genes in proximity of differentially expressed ir-lncRNAs.

**Additional file 2**: Differentially expressed lncRNAs in TCGA-BRCA

Additional file 3: Propagation walkscores of 127 proxy protein-coding genes in the TCGA-BRCA cohort.

**Additional file 4**: Canonical pathways, diseases and functions associated with the 127 proxy protein-coding genes.

**Additional file 5**: Spearman correlation coefficients of ir-lncRNAs with immune checkpoint expression in TCGA-BRCA.

**Additional file 6**: Prognostic value of ICR classifier across solid cancers. Forest plot showing HRs for death (overall survival) and corresponding 95%-confidence interval for the continuous ICR score and number of patients for each TCGA cancer cohort and RAQA breast cancer cohort. Significant positive HRs are visualized in blue and significant negative HRs are visualized in red. ICR enabled (HR < 1, p-value < 0.05) cancer types are indicated with orange asterisks and ICR disabled (HR > 1, p-value < 0.05) cancers are indicated with purple asterisks.

**Additional file 7**: Akaike information criterion (AIC), delta AIC, and HRs for death (overall survival) and corresponding 95%-confidence interval for ICR and 3 ir-lncRNA signature in 18 solid cancer TCGA datasets and RAQA cohort.

**Additional file 8**: Survival curves of 3 ir-lncRNA signature. Overall survival Kaplan-Meier curves in which the 3 ir-lncRNAs signature did not show any significant prognostic value. Dichotomization cutoff of ‘high’ (red) and ‘low’ (cyan) subgroups was based on the optimal cut-off point as determined by a 5-fold cross-validation analysis. Censor points are indicated by vertical lines. P-values were determined by logrank test.

**Additional file 9**: Correlation of 3 ir-lncRNA signature with immune subpopulations across tumor types. Pearson correlation heatmap between immune cell subpopulation enrichment scores and 3 ir-lncRNA scores.

## Data Availability

The TCGA RNAseq and clinical data analyzed during the current study are available at the GDC data portal, https://portal.gdc.cancer.gov/ or by using TCGA Assembler as detailed in [31]. The TCGA long non-coding expression profile data analyzed in this study are available at the TANRIC repository, https://ibl.mdanderson.org/tanric/_design/basic/download.html. The RNAseq raw data from the RAQA cohort are publicly available in the European Nucleotide Archive repository, https://identifiers.org/ena.embl:PRJEB41828 [65], and the mRNA expression profile and clinical data can be obtained from FigShare at https://doi.org/10.6084/m9.figshare.12901928 [66]. The lncRNA expression profile and the enrichment scores generated in this manuscript, in addition to the scripts used in this study can be found at https://doi.org/10.5281/zenodo.7092234 [67].

## Abbreviations

AIC: Akaike information criterion
AUC: Area under the curve
BLCA: Bladder urothelial carcinoma
BRCA: Breast cancer
CESC: Cervical squamous cell carcinoma and endocervical adenocarcinoma
COAD: Colon adenocarcinoma
Consensus^TME^: Consensus tumor microenvironment cell estimation
CTLA-4: Cytotoxic T-lymphocyte antigen number 4
ESTIMATE: Estimation of stromal and immune cells in malignant tumor tissues using expression data
GBM: Glioblastoma multiforme
HNSC: Head and neck squamous cell carcinoma
ICR: Immunologic constant of rejection
ICI: Immune checkpoint inhibitor
IPA: Ingenuity pathway analysis
KIHC: Kidney chromophobe
ir-lncRNA: Immune-related lncRNA;
KIRC: kidney renal clear cell carcinoma
KIRP: Kidney renal papillary cell carcinoma
LGG: Low grade glioma
LIMMA: Linear model for microarray analysis
LIHC: Liver hepatocellular carcinoma
lncRNA: Long non-coding RNA
LUAD: Lung adenocarcinoma
LUSC: Lung squamous cell carcinoma
OV: Ovarian serous cystadenocarcinoma
RAQA: Retrospective Arab Qatar cohort
READ: Rectum adenocarcinoma
RWR: Random walk with restart
SKCM: Skin cutaneous melanoma
ssGSEA: Single-sample gene set enrichment analysis
STAD: Stomach Adenocarcinoma
UCEC: Uterine corpus endometrial carcinoma
