# Supplementary figures and images for: Immune-related 3-lncRNA signature with prognostic connotation in a multi-cancer setting
Source: J Transl Med. 2022 Sep 30;20:442. doi: 10.1186/s12967-022-03654-7 (PMC9523969; doi:10.1186/s12967-022-03654-7)

**A.**

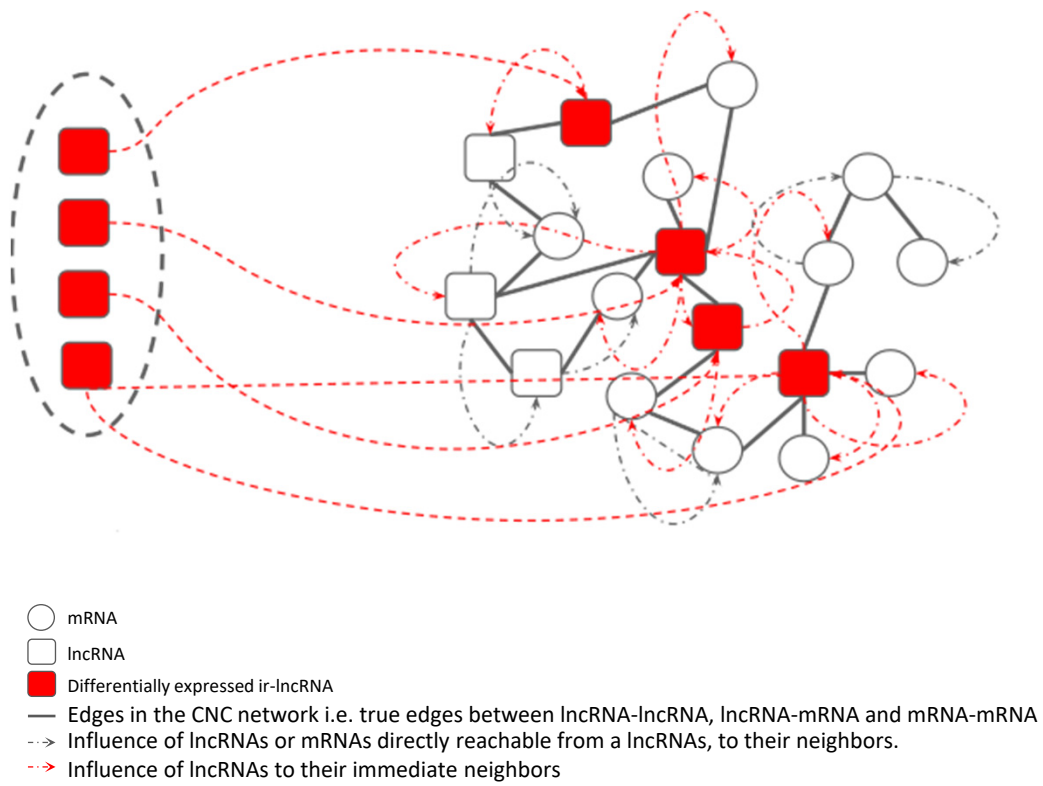

**B.**

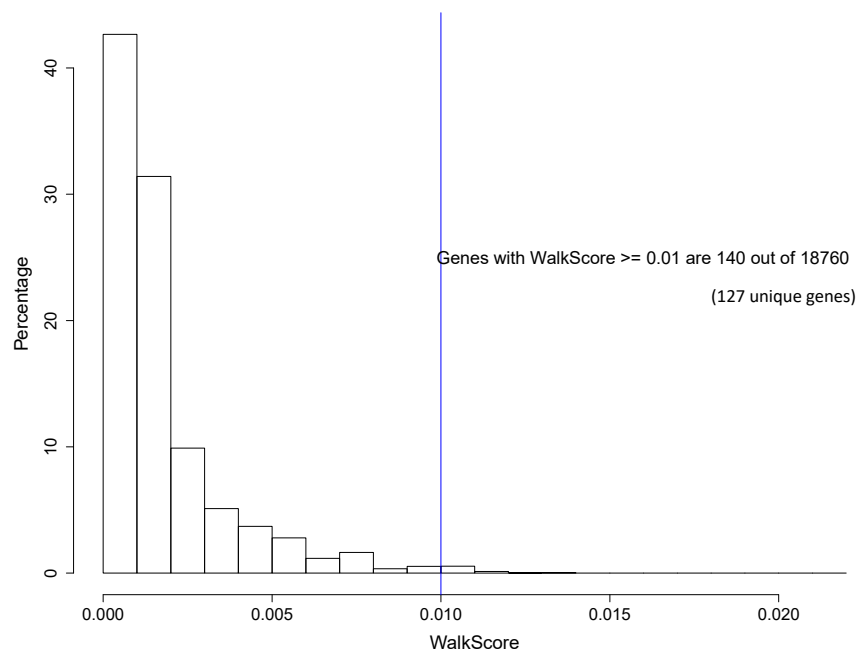

Supplement: Supplementary file 1 — Additional file 1: Mapping of differentially expressed ir-lncRNAs to protein coding genes. (A) Diagram representation of the random walk with restart global propagation network algorithm. (B) Walkscore distribution of protein-coding genes in TCGA-BRCA, with cutoff set at walkscore ≥ 0.01 to generate a ranked list of protein-coding genes in proximity of differentially expressed ir-lncRNAs. [file 12967_2022_3654_MOESM1_ESM.pdf]

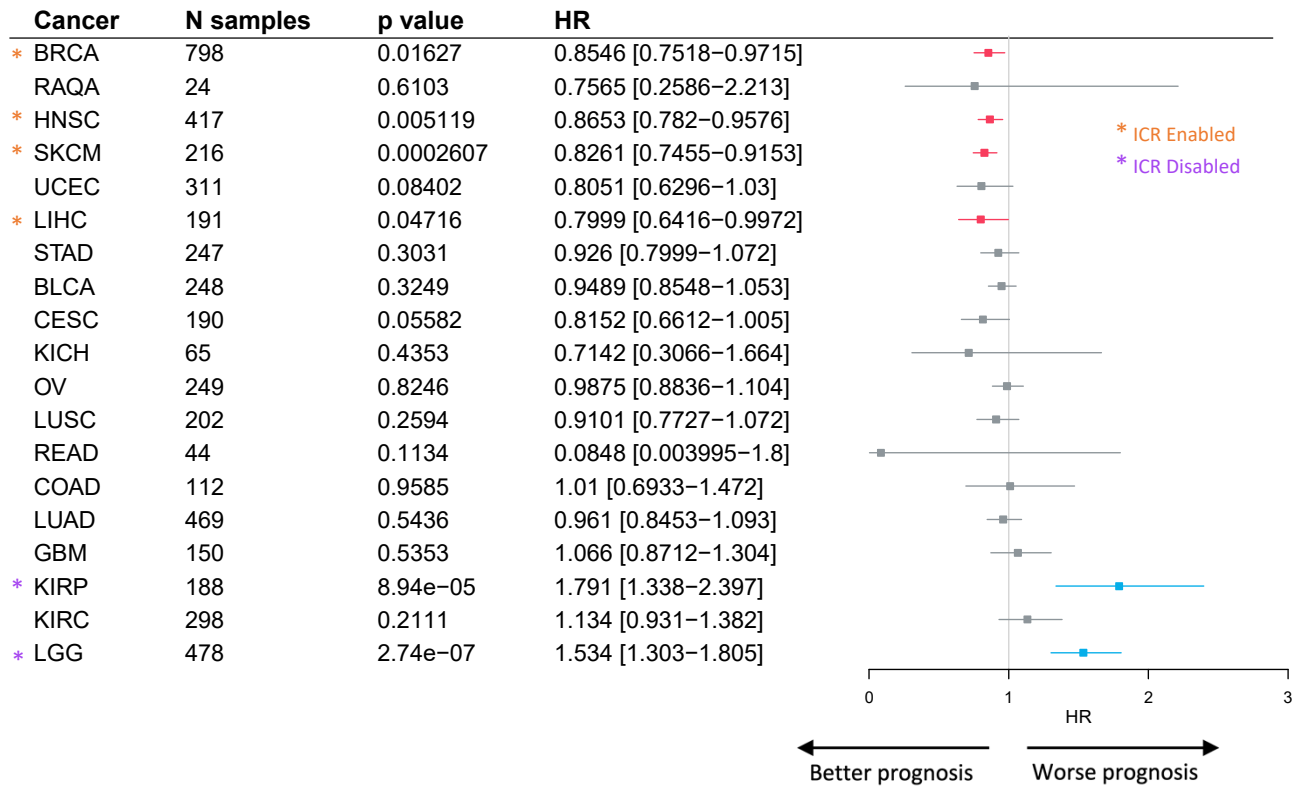

Supplement: Supplementary file 6 — Additional file 6: Prognostic value of ICR classifier across solid cancers. Forest plot showing HRs for death (overall survival) and corresponding 95%-confidence interval for the continuous ICR score and number of patients for each TCGA cancer cohort and RAQA breast cancer cohort. Significant positive HRs are visualized in blue and significant negative HRs are visualized in red. ICR enabled (HR < 1, p-value < 0.05) cancer types are indicated with orange asterisks and ICR disabled (HR > 1, p-value < 0.05) cancers are indicated with purple asterisks. [file 12967_2022_3654_MOESM6_ESM.pdf]

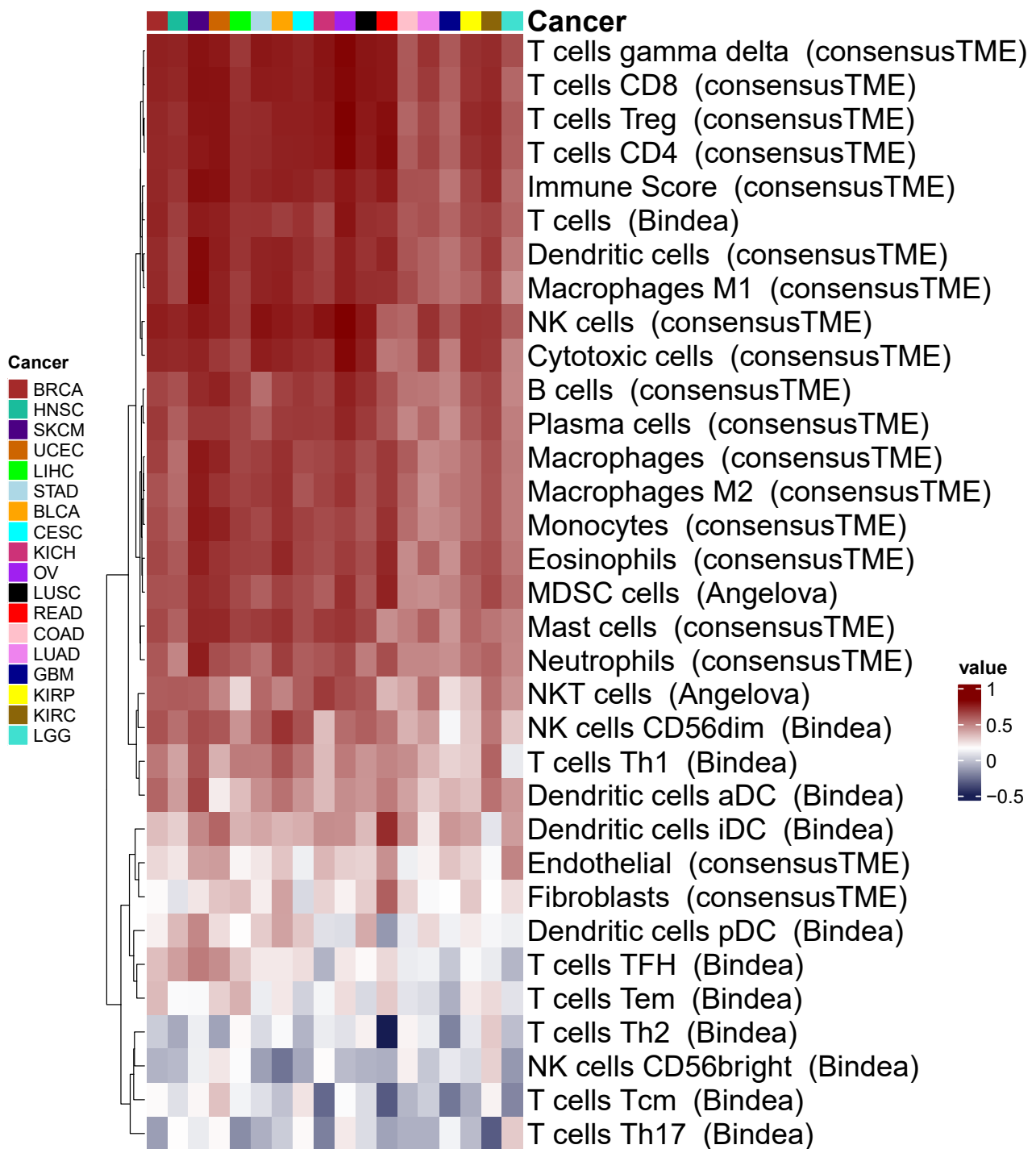

Supplement: Supplementary file 9 — Additional file 9: Correlation of 3 ir-lncRNA signature with immune subpopulations across tumor types. Pearson correlation heatmap between immune cell subpopulation enrichment scores and 3 ir-lncRNA scores. [file 12967_2022_3654_MOESM9_ESM.pdf]
