# Supplementary material for: Immune-related 3-lncRNA signature with prognostic connotation in a multi-cancer setting
Source: J Transl Med. 2022 Sep 30;20:442. doi: 10.1186/s12967-022-03654-7 (PMC9523969; doi:10.1186/s12967-022-03654-7)
Supplement: Supplementary file 8 — Additional file 8: Survival curves of 3 ir-lncRNA signature. Overall survival Kaplan-Meier curves in which the 3 ir-lncRNAs signature did not show any significant prognostic value. Dichotomization cutoff of ‘high’ (red) and ‘low’ (cyan) subgroups was based on the optimal cut-off point as determined by a 5-fold cross-validation analysis. Censor points are indicated by vertical lines. P-values were determined by logrank test. [file 12967_2022_3654_MOESM8_ESM.pdf]

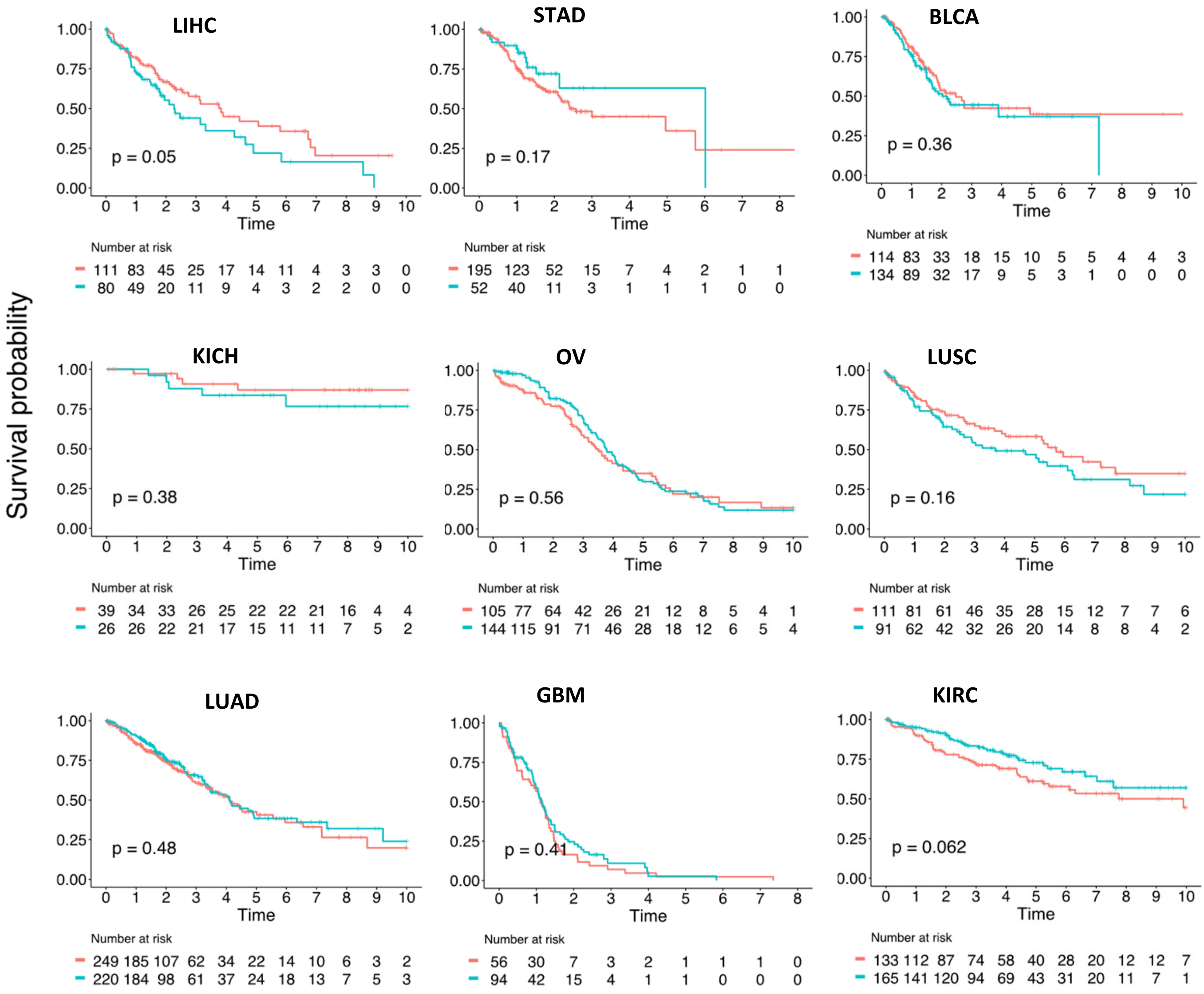
Survival probability
LIHC
p = 0.05
Time
Number at risk
111 83 45 25 17 14 11 4 3 3 0
80 49 20 11 9 4 3 2 2 0 0
STAD
p = 0.17
Time
Number at risk
195 123 52 15 7 4 2 1 1
52 40 11 3 1 1 1 0 0
BLCA
p = 0.36
Time
Number at risk
114 83 33 18 15 10 5 5 4 4 3
134 89 32 17 9 5 3 1 0 0 0
KICH
p = 0.38
Time
Number at risk
39 34 33 26 25 22 22 21 16 4 4
26 26 22 21 17 15 11 11 7 5 2
OV
p = 0.56
Time
Number at risk
105 77 64 42 26 21 12 8 5 4 1
144 115 91 71 46 28 18 12 6 5 4
LUSC
p = 0.16
Time
Number at risk
111 81 61 46 35 28 15 12 7 7 6
91 62 42 32 26 20 14 8 8 4 2
LUAD
p = 0.48
Time
Number at risk
249 185 107 62 34 22 14 10 6 3 2
220 184 98 61 37 24 18 13 7 5 3
GBM
p = 0.41
Time
Number at risk
56 30 7 3 2 1 1 1 0
94 42 15 4 1 1 0 0 0
KIRC
p = 0.062
Time
Number at risk
133 112 87 74 58 40 28 20 12 12 7
165 141 120 94 69 43 31 20 11 7 1
